# Understanding the stakeholders’ preferences on a mobile application to reduce door to balloon time in the management of ST-elevated myocardial infarction patients – a qualitative study

**DOI:** 10.1186/s12911-020-01219-6

**Published:** 2020-08-31

**Authors:** Nour Alkamel, Amr Jamal, Omar Alnobani, Mowafa Househ, Nasriah Zakaria, Mohammad Qawasmeh, Shabana Tharkar

**Affiliations:** 1 College of Medicine, King Saud University, Riyadh, Saudi Arabia; 2 Department of Family and Community Medicine, College of Medicine, King Saud University, Riyadh, Saudi Arabia; 3 Evidence-Based Healthcare and Knowledge Translation Research Chair, King Saud University, PO Box 90714, Riyadh, 11623 Saudi Arabia; 4 College of Science and Engineering, Hamad Bin Khalifa University, Doha, Qatar; 5 Medical Education Department, College of Medicine, King Saud University, Riyadh, Saudi Arabia; 6 Nursing Department, King Saud University Medical City, Riyadh, Saudi Arabia; 7 Prince Sattam Chair for Epidemiology and Public Health Research, Department of Family and Community Medicine, King Saud University, Riyadh, Saudi Arabia

**Keywords:** ST elevation myocardial infarction, Chest pain, Patient management, Mobile applications, Software, Cell phone, Qualitative research

## Abstract

**Background:**

ST-elevated myocardial infarction (STEMI) is a critical and time-sensitive emergency. The survival depends on prompt initiation of treatment requiring high precision and multi-level coordination between healthcare staff. The use of a mobile application may facilitate prompt management and shorten the door-to-balloon time by capturing information at the point of care and provide immediate feedback to all healthcare staff involved in STEMI management.

The objective of the present study has two primary components: (i) to explore the suggestions and opinions of stakeholders in the development of a novel mobile app for code activation in management of STEMI patients (ii) to find out the healthcare workers’ expectations including facilitating steps and challenges in the activation process of the proposed mobile app.

**Methods:**

Unstructured interviews were conducted with key informants (*n* = 2) to identify all stakeholders, who also helped in developing the interview protocol and prototype designs. In-depth, semi-structured, open-ended, face to face interviews were conducted on 22 stakeholders involved in managing STEMI patients. All interviews were recorded and transcribed verbatim. Data were analyzed using ATLAS.ti 8 software, allowing themes and subthemes to emerge.

**Results:**

The 22 participants included in the study were cardiology physicians (*n* = 3), emergency consultants (*n* = 4), emergency room (ER) senior nurses (*n* = 10), and cardiac catheterization lab staff (*n* = 5). The main themes identified during analysis were workflow and the App. The themes identified from the interviews surrounding the App were: 1) facilitating ideas 2) management steps needed 3) features 4) preferred code activation method 5) steps of integration 6) possible benefits of the App 7) barriers and 8) possible solutions to the suggested barriers. Most of the interviewed stakeholders expressed their acceptance after viewing the proposed mobile app prototype.

**Conclusion:**

The study identified the mandatory features and the management steps needed from the stakeholder’s perspectives. The steps for integrating the current paper-based workflow with the suggested mobile app were identified. The expected benefits of the App may include improved and faster management, accuracy, better communication, and improvement in data quality. Moreover, the possible barriers might comprise of doubtful acceptability, device-related issues, and time and data-related challenges.

## Background

ST elevated Myocardial Infarction (STEMI) is usually a life-threatening and time-sensitive critical emergency necessitating prompt management by reperfusion therapy and percutaneous coronary artery intervention (PCI) [1]. American College of Cardiology and American Heart Association (ACC/AHA) guidelines recommend a time interval of not more than 90 min for improved patient survival and outcomes, called door-to-balloon time (D2B) [2]. To state briefly, the incremental split would be ideal if STEMI diagnosis is made from the start of symptoms within 30 min (door to Electrocardiogram (ECG) time), the cardiac team mobilized with patients arrival at catheterization (cath) lab in the subsequent 30 min (ECG to cath lab time), and the blocked artery is opened in the last 30 min (cath lab arrival to device time) [3]. Studies have suggested shorter D2B time to be associated with improved survival outcomes, reduced one-year readmission rates and lower healthcare costs [4–6]. Extreme emphasis has been made to initiate immediate treatment following diagnosis as every minute delay damages the already hypoxic heart muscles [7].

In the US, progressive advancements in health care systems with a focus on adherence to the detailed clinical guidelines have reduced the D2B time form > 113 min in 2003 to 76 min in 2008 [8]. An earlier study from Saudi Arabia reported a mere 65 and 42% of male and female cardiac patients with myocardial infarction (MI) undergo treatment within D2B time respectively [9]. Although national statistics are not available, data from such sporadic studies suggests a delay in seeking medical attention. Diagnosis of MI depends on the patient’s symptoms upon presentation, raised levels of troponin and other biomarkers of myocardial necrosis, and typical ECG changes showing elevated ST-segment [10]. Prompt initiation of treatment within the door to ECG time and especially the ECG to Cath lab time results in shorter D2B time with marked improvement in prognosis [11, 12]. Door to ECG time depends on the patient utilization of Emergency Medical Services (EMS) and swift arrival at the hospital while hastening the ECG to cath lab time depends on large-scale and rapid coordination between various healthcare personnel including nurses, cath lab technologists, radiologists, emergency physicians and cardiac surgeons. Data from the Gulf registry have reported patient delays in using EMS services, thereby exerting enormous pressure on the healthcare team [13]. The success of the STEMI management depends on highly coordinated efforts on collecting and verifying the required data. The current protocol of STEMI activation necessitates multiple steps before the cardiology physician can respond to the code alert [14]. Incomplete or delayed tasks have shown to adversely affect the quality of healthcare in addition to the risks involved from paper-based checklists [15]. Although checklists have proven to enhance medical care and improve the quality of care [16], hand-written checklists are not always flawless, thereby compromising the management and prognosis. However, with the advent of the digitalization of checklists by electronic-based systems, it was possible to overcome the deficiencies mentioned above but with considerable inconvenience due to reliance on fixed desktops when the patients had to be mobilized for various other diagnostic tests [17]. Real-time performance feedback to the healthcare team during STEMI activation is associated with a shorter door-to-balloon time [18–21]. Besides, STEMI code activation can be done promptly, without having to progress through routine steps or being blocked by the physical boundaries of the hospital (e.g., the cardiology physician can respond and agree to activate the STEMI code even from outside the hospital).

Research studies have demonstrated the effectiveness of using mobile applications and instant messaging for faster and accurate inter-team communication facilitating emergency alert [22, 23].

For instance, the use of instant messaging smartphone applications like WhatsApp as a communication medium for transfer of health information between the healthcare care personnel has demonstrated efficiency in handling obstetrics and neonatal care services.

Similar applications have proven superior efficiency in attending emergency injury response. Eksert and his colleagues demonstrated encouraging results in managing acute injury, accident and combat cases by using such applications that have largely reduced workload and time loss resulting in better coordination of the emergency response team and improved quality of care.

Likewise, STEMI management team obligates dealing with acute emergency response demanding simultaneous and multi-coordinated efforts that can be simplified by customized apps for STEMI management. Recent studies showed that the use of mobile applications can support shared decision making, empower patients, and can increase patients’ satisfaction. Mobile applications can also save health care providers time and increase their work efficiency [24].

A mobile app could also capture information at the point of care, provide immediate feedback to all healthcare staff involved in STEMI management, and facilitate data retrieval for statistical purposes. Also, the mobile app might reduce the false-positive activation. However, there is a need to analyze the optimal approach to the solution by an in-depth assessment of step by step procedures.

With this background, the current study was conducted to qualitatively explore the opinions and suggestions of healthcare workers in the development of a mobile-based application for code activation in the management of patients with acute MI. The facilitating steps and time-related challenges in the initial activation and management of STEMI were also explored. To our knowledge, this is the first study from Saudi Arabia, to explore the use of mobile applications in STEMI management and the results would prove highly beneficial to the healthcare personnel, policymakers and decision-makers in evolving improved strategies for a better quality of care.

## Methods

### Study design and sampling

A qualitative design was adopted with the use of in-depth, semi-structured, open-ended, face to face interviews [25] incorporating a semi-structured guide. This method is the most suitable to be applied in order to explore suggestions and opinions from the stakeholders as well as to understand healthcare workers’ expectations. Qualitative methodology allows naturalistic inquiry to elicit humans’ feelings and thoughts [26]. A single researcher was responsible for data collection during the period 4th February to 19th March 2019. The stakeholders were identified by analyzing clinical practice guidelines and the current workflow applied at the selected Center through unstructured interviews with key informants (ER manager and the head of Catheterization Laboratory). These key informants also helped in developing the interview protocol and prototype designs. This process of building interview protocol aligned with what were suggested by [26, 27]. Purposive sampling technique with maximum variation sampling from all stakeholders who participated in the management of STEMI patients was applied to understand the issue from multiple perspectives. All interviews were confidential and coded during transcription to ensure anonymity.

### Data collection

Interview is defined as “purposeful conversation” [25] and it is the well-known qualitative method to elicit information from informants [26, 27]. Interview method is a primary source of data and able to explore people’s feelings and thoughts [28]. Interviews can be done one to one or group interviews and can be conducted using face to face, email and online chat [28].

All interviews were conducted in English at the hospital site. Collection of data continued until saturation between different groups was reached. In this study, our sample size reached 22 participants, which suffices the stated minimum number of participants to be at least 15 people [29]. All interviews were audio-recorded after receiving the permission from the interviewees, and these recordings were later transcribed. The study objectives were clearly explained before the in-depth interviews.

### Interview components

The interviewees were asked to describe the daily management of STEMI patients, followed by a discussion on facilitators and challenges in the treatment pathway, which led to the identification of areas that needed improvement. Then, the possibility of a future mobile app was explored, with a focus on management steps, and the process of integration between the current paper-based workflow and the proposed mobile app, the concerns and barriers related to the application of App in STEMI management. Stakeholders were requested to suggest strategies to overcome the expected barriers. Triangulation was achieved by exploring the current workflow, its pros and cons from multiple resources and multiple personnel involving the key informants and the stakeholders who have different perspectives and roles in STEMI management. Based on the identified needs through key informant’s interviews, the current practice of STEMI management and universal guidelines prototypes were designed. The prototypes encompassed multiple interfaces, including ER nurse, ER consultant, catheterization and time interfaces.

### Analysis

The field notes of the interviewees’ responses were also documented. All the interviews were transcribed using Microsoft Word for analysis by the same person who conducted the interviews. Transcripts coding was done using ATLAS.ti 8 software, which helped in organizing codes and retrieving the relevant quotations. Codes were created for each sentence based on the objective, followed by the grouping of codes to form significant themes based on links between codes. Early coded transcripts were reviewed for consistency and the emerging themes were discussed later by the research team. The disagreement was resolved by discussion until consensus was reached. Two external clinicians reviewed the final themes and sub-themes. The inter-coder reliability was 87 and 80%, respectively.

### Ethics approval

The study was approved by the Institutional Review Board of the College of Medicine, King Saud University with the following IRB No: E-17-2753. All participants provided their written consent before participation in the interview.

## Results

Twenty-two interviews were performed with the stakeholders who participated in the STEMI management at the hospital (Table 1).

**Table 1:** Interviewee code and characteristics

Interviewee code	Age group	Position	Experience (years)
*Nurse 1*	40–49	Staff nurse	17
*Nurse 2*	50–59	Staff Nurse	31
*Nurse 3*	30–39	Staff Nurse	9
*Nurse 4*	40–49	Charge nurse	19
*Nurse 5*	40–49	ER manager nurse	23
*Nurse 6*	30–39	Staff Nurse	16
*Nurse 7*	40–49	Staff Nurse	18
*Nurse 8*	30–39	Staff Nurse	13
*Nurse 9*	30–39	Staff Nurse	7
*Nurse 10*	30–39	Staff Nurse	9
*Cath 1*	30–39	Cath staff	10
*Cath 2*	30–39	Cath staff	8
*Cath 3*	30–39	Cath staff	10
*Cath 4*	30–39	Cath staff	10
*Cath 5*	30–39	Cath Lab. Charge nurse	10
*Physician 1*	50–59	ER consultant	27
*Physician 2*	30–39	ER consultant	15
*Physician 3*	30–39	ER consultant	10
*Physician 4*	30–39	ER chairman	11
*Cardiologist 1*	30–39	Cardiology consultant	13
*Cardiologist 2*	40–49	Senior specialist	18
*Cardiologist 3*	60–69	Cardiology consultant	38

The central themes and subthemes that emerged from the interviews were mostly regarding App benefits, barriers, and solutions to possible barriers, facilitating ideas, App features, steps in integration, App concerns and needed App management steps.

### Challenges related to the current paper-based workflow

The study identified specific challenges in STEMI patient management at the hospital which can be grouped in the following themes in a nutshell; a) delayed diagnosis: due to atypical symptoms (grey zone) and awaiting cardiac specialist to review ECG. b) ECG is paper-based and not integrated with the hospital’s electronic medical record (EMR), forcing the physicians to communicate the ECG images on WhatsApp. c) Limited resources on weekends and after working hours cause a delay. Moreover, with a single ECG machine at the emergency department delays are not infrequent if there are more patients with complaints of chest pain. d) Patient-related delays in arrival time. e) Process related delays concerning the failure of digitalizing the current paper-based workflow is another challenge affecting data accuracy and retrieval.

Based on the identification of the challenges faced by the workforce in action, some suggestions that were provided include; a) provision of more ECGs with linkage facilities to EMR. b) Improving communication between the ER consultant and cardiology team, improving the code activation process through an automatic, unified call for on-call team members c) increasing patient awareness on the use of EMS systems.

### Thoughts on the implementation of a new mobile application

#### App: facilitating ideas

The stakeholders were prompted to think about how to implement their ideas to facilitate the workflow through the use of the mobile app. Some of the interviewees suggested activating the code simultaneously to all relevant stakeholders through an exclusive App. It is illustrated in the following quote:

*“In a mobile application, if a patient arrives and they are activating this mobile application, then it will be a better idea so that everybody can get the same signal at the same time.”* (Cath 3).

The other suggestion made by six other respondents was concerning developing a sophisticated mobile app by linking to red-crescent to enable early pre-hospital activation. One interviewee suggested the following:

*“Mobile application will help too much for pre-hospital to the hospital, and it will help in communication regarding the cases that we did not decide in emergency consultant activate or not to activate you need the availing of the interventional cardiologist and the primary contact in the mobile application.”* (Physician 1).

Some interviewees suggested using a particular mobile app to send the ECG report, which enables prompt communication with the cardiology team. Other interviewees proposed to use an App to show a real-time for each management step as quoted below:

*“I think more on like timings. Actually, we are using a stopwatch it is not really helpful at all because this stopwatch actually different timing, so we are still looking for a real-time this time, is the time for mobile. So we are going to make an App it is mostly for the timing it helps to lesson timings, I do not know.”* (Cath1).

One ER consultant suggested creating an algorithm through the App to help identify high-risk patients, including the ability to analyze ECG, while another cardiac consultant suggested having a checklist involving major questions regarding STEMI cases. The actual comment is as follows:

*“If there is a checklist of 10 things that we have to do in the Emergency Room (ER) maybe, it will take 1 minute or a couple of minutes this will help a lot I think to also exclude other serious diagnosis and will help also us to make the plan for STEMI.”* (Cardiologist 1).

#### App management steps needed

**Fig. 1:**
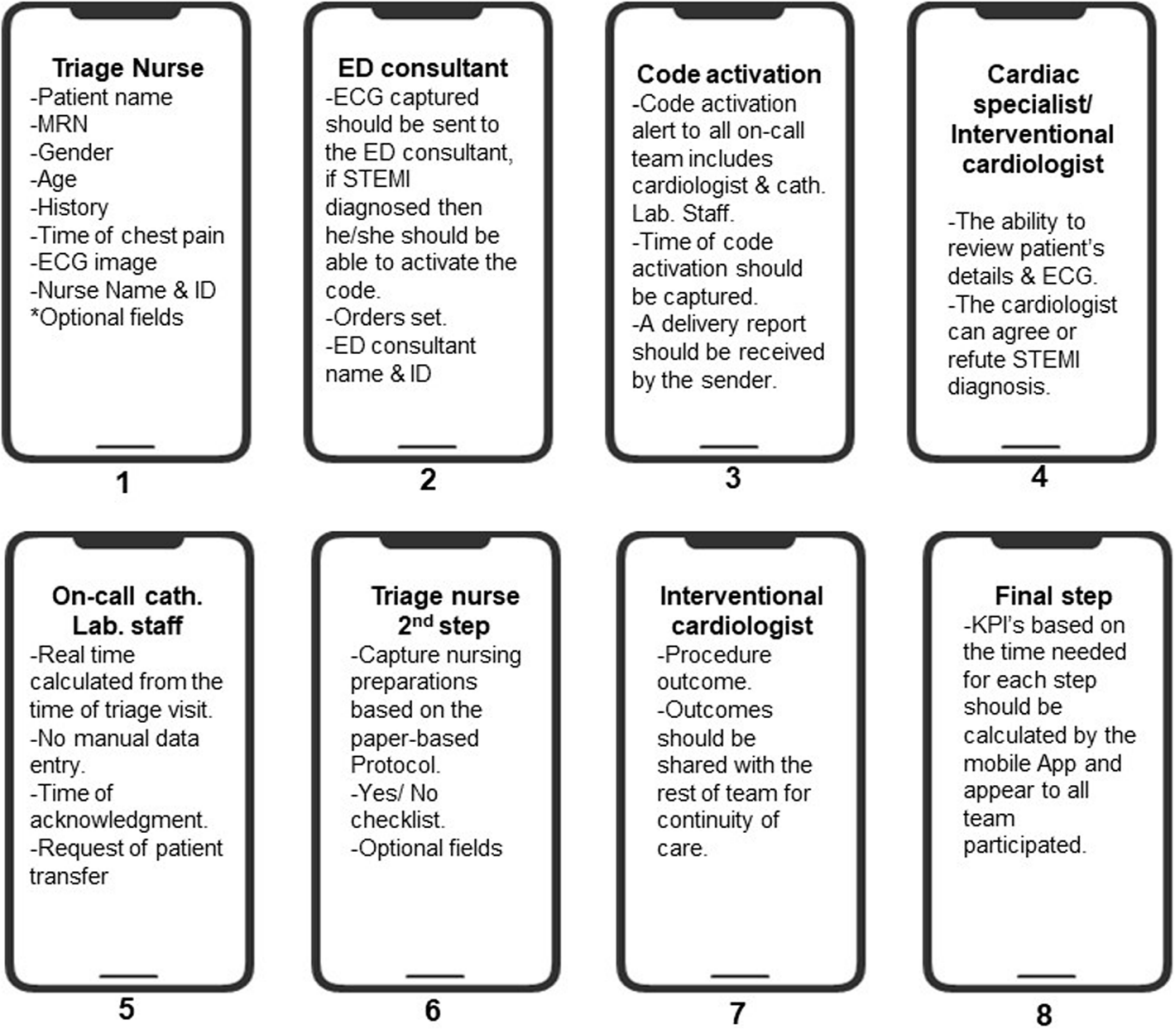
Mandatory features within each management step

Management steps needed from the STEMI mobile app are summarized in Table 2. The main features needed for each step are also presented in Fig. 1.

**Table 2:** Management steps needed from STEMI app

Management steps needed	
Each stakeholder group has a specific interface include their tasks only separated from the rest.	
Ability to send high-quality ECG.	
Ability to capture time of each step automatically.	
Ability to modify previously entered data.	
Include the same information available on the paper-based protocol.	
Integrated with the electronic medical record.	
Code activation through the App.	
Code activation delivery report.	
Calculating the time needed for each management step automatically.

#### Features to be added to the app

Stakeholders suggested some features that might add value to the App, such as adding the time of arrival of each team member once the code is activated for accuracy. A code cancellation option was also suggested.

Another ER consultant suggested adding some criteria to analyze the ECG using the App itself and to include a toolbar and ruler for the ECG image. He also suggested adding the ability to retrieve old ECGs – if any existed – for comparison. The following quote illustrates this:

“*… is there any way to put like, I do not know, its very difficult but you have like ECG captured by the program, so let’s say this patient came like in the weekend, as some ECG changes resemble STEMI. And today, he came again. Is there any way to retrieve both ECG done like a week ago and this is today for comparison.”* (Physician 2).

The use a different alarm tone, if the patient status was critical, was also suggested, as illustrated in the following quote:

*“And if your patient is critical use different code alert as if the patient is in hypotensive shock.”* (Cath 5).

#### Preferred code activation method

Stakeholders were asked about their preferences for code activation using the mobile app, and their responses show that 45% preferred direct call, 36% favored alarm, while 14% preferred both methods for information delivery. Those who preferred an alarm focused on the importance of receiving a report of the alert.

#### Steps of integration

Stakeholders suggested many steps to integrate the current paper-based workflow to the proposed mobile app. First, each user should be provided with credentials to use the App. The proposed App should be composed of several phases for each user group; ER, cardiology, and Cath Lab phases. All users should be trained to use the App and policies and procedures should be developed for that purpose. Time in the App, EMR, and ER computers should be matched to prevent any discrepancies. The paper-based protocol should be converted to an electronic version. Finally, a pilot study on a few numbers of patients should be applied before going live.

#### App possible benefits

Several stakeholders mentioned the management process benefits of using a STEMI app that would facilitate diagnosis and shorten door-to-balloon time. Two statements from the stakeholders illustrate these benefits:

“*… so easy to diagnose.”* (Nurse 7).

“*… shorten the time to activation, door-to-balloon.”* (Nurse 9).

“*… retrieval is better. This could help me, later on, to review the case if there is a teaching point, so yes, it is helpful, this is added value.”* (Physician 3).

*“Better documentation.”* (Physician 1).

Communication was also reported a possible benefit, especially after working hours. One cath staff commented:

*“Sometimes we are at home, so this is double calling, but if the application can show they received the activation and acknowledged the code then that can help, we do not need to call, we need to run.”* (Cath 5).

Other stakeholder responses suggested that using this App will lead to accurate diagnosis and documentation and reduce the false activation rate. The following comment illustrates this:

*“So, the entire process will be more accurate.”* (Physician 3).

#### App barriers

Stakeholders identified many possible barriers related to the use of a STEMI management App. The primary barrier reported in this study were device-related issues, including range, technical errors, and the internet. The following statement captured this barrier:

“*… .technical errors, errors of the mobile application, and I think the internet.”* (Cath 1).

The other barrier reported by some stakeholders was time-consuming. As explained by one of the emergency consultants:

*“Our system gets complicated when EMR was introduced, so adding an App will impact the time negatively.”* (Physician 4).

The acceptability of using this App, especially from old physicians, was also mentioned.

*“Maybe some people will not be familiar with them [Apps] to start so it might take some time to be familiar with that, especially senior people, sometimes they take a longer time to use it.”*

Another barrier retrieval of previously entered data, which stated as;

*“Maybe we cannot revise the data like if we are getting the activation of STEMI through that mobile application, we will get an idea of the patient and the management part initially. But through that mobile system, maybe we will not revert back.”* (Cath 3).

Feasibility also was reported as a barrier by one of the nurses:

*“For the eyeball staff maybe it will be difficult comparing to the number of patients that we see.”* (Nurse 1).

Only one physician reported his worry of missing query STEMI cases “grey zone” if they relied on the proposed App. This physician commented:

*“Sometimes we have a grey zone in the ECG, so if you depend too much on that application for code activation, you might miss those many patients in the grey* zone.”

#### Possible solutions to the app barriers

Table 3 offers suggestions made by the stakeholders to overcome the possible App barriers.

**Table 3:** Solutions to the App barriers

Barrier	Possible solutions
Device-related	Use an appropriate screen size. Use a pocket-size device for nurses so they can carry it all the times. Provide internet for all devices used. Run the App offline. Provide sufficient devices. Have spare batteries. Use a unique and high-volume ringtone for code activation and assign different tones to different tasks.
Time-consuming	Using a bar-code reader to register patients in the App and capture their name, medical registration number (MRN), and age. Update stakeholders information daily so it will be captured automatically once the user logs in.
Acceptability	Orientation and good training. Regular meetings so everybody is knowledgeable.
Data	Have a backup service. Have another standby plan for code activation. Restrict App access to the authorized staff only. Include a data retrieval function. Code patient data when transmitting information to the team. Provide each user with a password and request it to be changed every three months. Have the ability to review who has access to patient data.
Missing query cases	The ER consultant is the one who should receive the ECG, not interns or residents. The ER consultant should communicate immediately for query cases.

#### Uncertainty about the application

About 55% of the participants (12 out of 22) expressed their uncertainty toward App use in STEMI management, however, after showing the prototypes, they expressed their acceptance and were enthusiastic to see it in reality which was reflected in the following comment:

*“I think the emergency physician is willing to use these new … let us say modalities or technologies to facilitate patient care.”* (Physician 3).

## Discussion

This qualitative study facilitated detailed accounts of stakeholders’ opinions and recorded the suggestions on the development of a mobile-based application in the management of STEMI patients. The present study identified the management steps necessary for the App to provide better quality care with reduced patient management time. The stakeholders opined that the steps be included separately in phases for each stakeholder’s specialty according to his or her role in STEMI management. More importantly, calculating the time required for each completed task and this management step is considered to be crucial to stakeholders since the data are used as key performance indicators (KPIs) that reflect the quality of the stakeholders performance.

The other key findings are enumerated below;

### Mandatory features to be included in app

The stakeholders suggested certain specific characteristics that were deemed mandatory in the App which includes, patient demographics, a brief history of the patient, risk factors, and an ECG image. However, simplicity and minimal time consumption are the two priorities to be considered while designing the App. More information collected will lead to a more accurate diagnosis but may also increase the time taken for the code to be activated. Furthermore, the information should then be sent to the ER consultant, and a code activation feature granted to the ER consultant sounds a unified call or single alarm to on-call team members [30]. Likewise, an important feature frequently noted by stakeholders was the ability to receive a notification delivery report from each team member to confirm the receipt of code alarm in order to ensure that the entire team has received the message and alert emergency staff. Another important area that needs to be considered is the confirmation of the STEMI code receipt by the cardiologist, particularly for the query cases. Other desirable features include capturing the name and ID of each user, making any entry and calculating a timestamp for each step performed using the App. This protocol can ensure accurate documentation, save time, and make it possible to track who accessed the App or made an entry.

### Steps of integration from paper-based model to digital type

A phased conversion was suggested in a simplified manner integrating EMR, ER and Cardiac team followed by training. The primary concept suggested by most of the participants was the ability to exchange information between the proposed App and EMR which is crucial in saving time. Galligioni and his team made similar suggestions to integrate m-health in oncology [31]. The mobile app linkage with EMR assists in the preview of all the relevant information related to the patient, however, the interviewees felt the lab tests difficult to document in the mobile App due to time constraints.

### App benefits over paper-based workflow

Based on semi-structured interviews with 22 stakeholders, the study after viewing prototypes found that the benefits of the App outweighed the current paper-based workflow in the management of STEMI patients. The expected benefits included timing accuracy, better communication, improved and faster management, and improvements in data quality, which are in accordance with similar studies [32, 33] . These findings were not surprising because the current workflow is based on a paper-based protocol that can be easily lost or contain inaccuracies, and the current code activation depends on the consecutive calling of all team members, which is also time-consuming.

### App barriers and challenges

Despite the benefits, many barriers were predicted while using the mobile app in such a domain. These barriers included acceptability, device-related issues, and time and data-related challenges, which is consistent with other research [34–36] . The majority of interviewees expressed concern about relying on a mobile device to activate the STEMI code because any error, delay, or hanging in response time might result in a significant delay.

To the best of our knowledge, this study is the first in Saudi Arabia to investigate stakeholders’ preferences for a mobile app for the management of STEMI patients. This study provided an overview of stakeholders’ expectations and indicated areas for improvement and considerations that need to be taken into account for the design of such an App. Owing to its novelty, it is hard to compare the present study with similar literature from regional or global context. However, a small number of studies have demonstrated the effectiveness of integrating mobile applications in healthcare delivery. Apps like Diagnosaurus, LabPro values, Archimedes, MedCalc, AHRQ ePSS are some of the common apps effectively used by healthcare professionals for.

patient management and monitoring [37]. Other studies have tested Apps like Siilo and WhatsApp for effectiveness in instant communication and consulting during patient management demonstrating faster and efficient mode of contact for the caregivers [38, 39]. Furthermore, clinical monitoring Apps, intensive care units Apps, decision-making Apps in radiology units have together revolutionized digital healthcare facilitating quicker communication and improved patient management [40–42]. The functionalities of the proposed naive STEMI App follow an integration of the aforementioned components. The consolidated App may encompass features of communication exchanges and high alert signaling, in addition to patient management and monitoring.

#### Study limitations

The study contains some limitations. First, this study used prototypes and did not refer stakeholders to an actual App. Therefore, the results might reflect on the personal preferences and expectations, and there may be a disparity with the actual trial. Second, a convenience sample of stakeholders who manage STEMI patients was used. Significant variations in the study interviewees were followed. However, reliability was attained by data triangulation because the data were collected from different resources and stakeholder perspectives. Study themes and subthemes were validated by two external clinicians with experience in the field of cardiology.

The stakeholders’ responses and needs reported in this study were influenced by the current workflow and resources at the hospital. Therefore, the findings cannot be generalized to other settings.

#### Research implications

The research implications point towards novel use of the innovative technologies in healthcare and emergency medical services to augment better patient management and improve patient outcomes. Management of STEM-I specifically requires high precision coordinated efforts by multidisciplinary team in shortest possible time to improve patient survival and reduce associated morbidity and mortality. The study findings may guide App developers and entrepreneurs in developing the novel App mandatory features with STEMI management steps included in it. Given that time-management is a significant factor in STEMI treatment, these findings may prove pivotal for cardiologists, ER physicians, nurses, and cath. Lab staff who may integrate STEMI App into their management plan. The use of the mobile application thus not only quickens the EMS utilization, in addition, it also enables each other to know the status of teamwork and feasibility in communications exchange and retrieval of information. Adoption of the App by policymakers may reduce the valuable time to cover the prehospital STEMI patients’ management thereby advancing other phases of management with a high probability of saving patient’s life.

## Conclusion

The main findings point towards the identification of mandatory features and the management steps needed as principal suggestions of the stakeholders for the proposed mobile application. The steps leading to integration between the current paper-based workflow and the suggested mobile app were also identified. The possible benefits of using such an App may include accuracy, better communication, improved and faster management, and improvement in data quality. And the possible barriers suggested were questionable acceptability, device-related issues, time and data-related challenges.

## Data Availability

The datasets used and/or analyzed during the current study are available from the corresponding author on reasonable request.

## Abbreviations

*ACC / AHA*: American College of Cardiology / American Heart Association
App: Application
*ECG*: Electrocardiogram
*EMR*: Electronic Medical Record
*ER*: Emergency Room
*EMS*: Emergency Medical Services
*IRB*: Institutional Review Board
KPI: Key Performance Indicator
*MRN*: Medical Registration Number
*MI*: Myocardial Infarction
*PCI*: Percutaneous coronary artery intervention
*STEMI*: ST-elevated myocardial infarction
